# Risk of eye infections in dental personnel and the need for its prevention: a case report

**DOI:** 10.1186/s12348-020-00211-5

**Published:** 2020-08-27

**Authors:** Ritvi Arvind, M. Roma

**Affiliations:** 1 Department of Conservative dentistry and Endodontics, Manipal College of Dental Sciences, Mangalore, India; 2 Manipal Academy of Higher Education, Manipal, India

**Keywords:** Eye infection, Protective equipment, Dental settings, Prevention

## Abstract

A lot of dentists and dental personnel are at high risk of contracting eye infections during operative procedures involving aerosols. As many may not be aware of it, they often ignore the precautions to be taken for prevention of such infections. This is one such case report of a dental intern where an eyelid infection arose shortly after she treated a patient with an infected tooth in an operative procedure. This case report emphasizes the importance of preventive barriers for the dentist, and that how special protective gear is required for doing restorative cases which involve dealing with infection.

## Introduction

Every occupation has its own risks and benefits, and safety concerns are of paramount importance. Protection with proper safeguard is mandatory. With the advanced knowledge about infection control and personal protection, a lot of emphasis is required on eye protection.

Frequently executed restorative and operative procedures like caries excavation, restorations, oral prophylaxis, etc. are performed using high power-driven handpieces. During the dental procedures, a lot of particulate matter like spicules of caries, calculus, amalgam, blood etc. gets released which may get lodged into tissues. The working handpiece generates lot of aerosols which carry an array of microorganisms producing infections to respiratory tract, eyes, skin, etc.

Eye infections can arise due to a variety of organisms (bacterial, viral, fungal, helminths) and its severity can range from mild swelling of the eyelid to complete blindness. Dentistry is one of the professions which is highly risked for ocular infections on a routine basis [1]. Dental professionals are bound to take necessary precautions to prevent eye related injuries [2]. Previously cases have been reported which have shown a connection between dental treatment and ocular irritation [3, 4]. Tremendous effort has to be created between the medical and dental professionals to understand the need for eye care. Certain National safety agencies, like Occupational Safety and Health Administration (OSHA), American National Standard Institute (ANSI), Centers for Disease Control and Prevention (CDC), American Dental Association (ADA) have set prompt guidelines for the proper usage of infection control measures and personal protective equipment (PPE) [5].

This case report delineates the relationship between ocular infections secondary to allergic reaction due to dental treatment among the dentists. This article aims at improving the knowledge of eye related injuries among the dental fraternity while emphasizing the need for protective measures.

## Case report

**Fig. 1:**
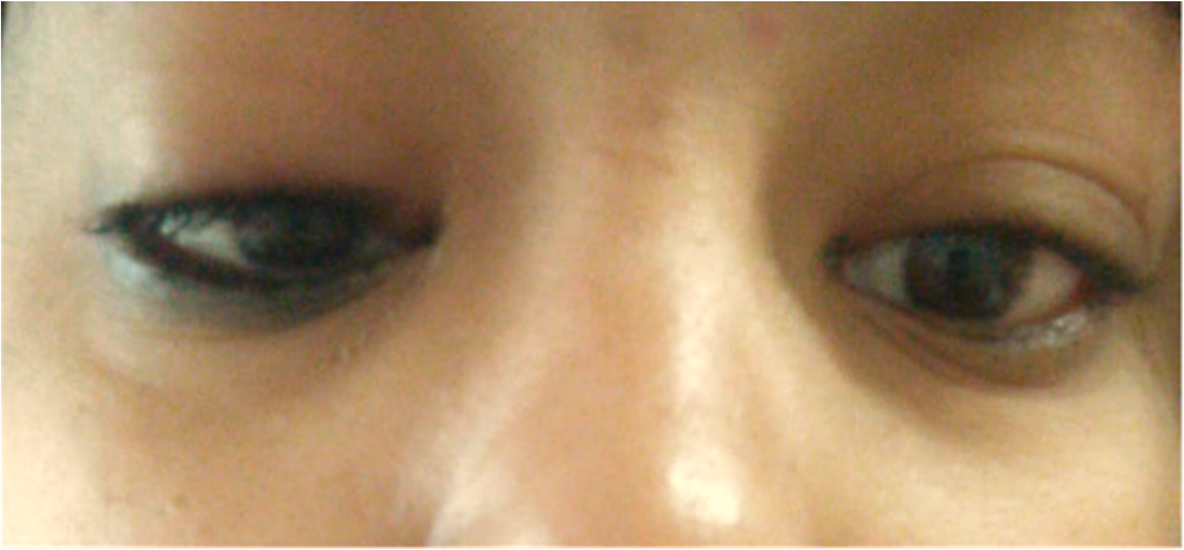
View of the eye showing diffuse swelling of the upper lid of the right eye with a normal anterior segment and mild congestion in the left eye, 3 days after performing the dental procedure

**Fig. 2:**
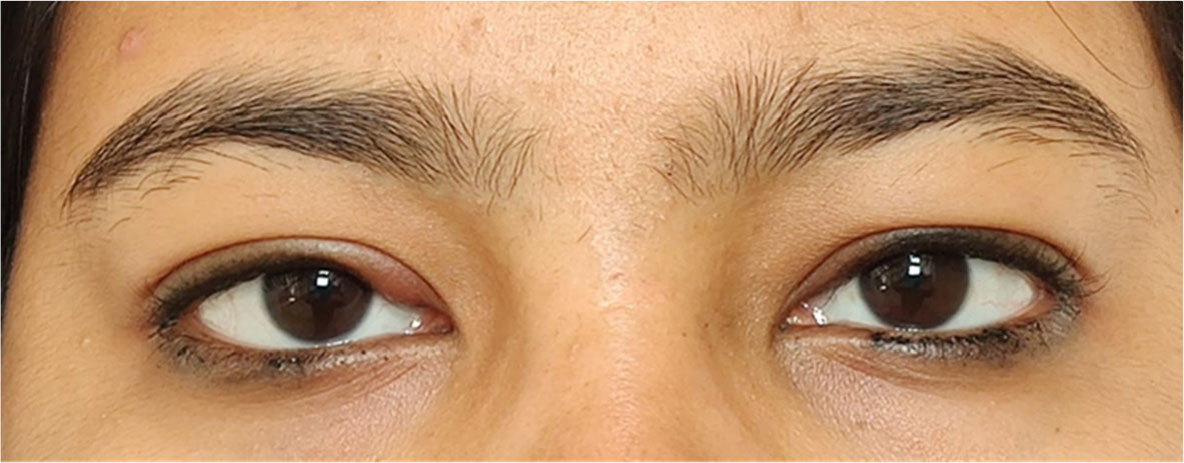
View of the eye showing nodular swelling at the medial margin of the upper lid of the right eye with normal anterior segment after 1 week of performing the dental procedure (Recurrent infection)

A 22-year old female dentist had treated a patient, with deep caries management under rubber dam isolation. During the treatment, the dentist got injured with a spicule from the cavity in the right eye. She rinsed her eyes with the water couple of times. In spite of regularly rinsing, the dentist developed irritation and foreign body sensation in the right eye immediately after the procedure. Three days later, a full-blown infection was noticed. She developed redness, pain, inability to fully open the eye without discomfort, yellowish discharge which often sealed the eye shut during sleeping, and generalized malaise. The right eye had diffuse swelling of the upper lid with a normal anterior segment and mild pain and difficulty in opening (Fig. 1). Mild congestion was seen in the left eye (Fig. 1) and then she visited the Ophthalmology department. Upon consultation, the ophthalmologist performed slit lamp examination and was diagnosed with Bacterial Blepharitis. There was no blurring of vision, no dilation of pupils, and no abnormality was detected in the adjoining structures. She was prescribed antibiotic ointment (Moxigram, Moxifloxacin Hydrochloride 0.5% w/v three times a day for a week) with topical eye drops (Toba, Tobramycin Ophthamic solution, 0.3% w/v four times a day for 1 week) and normal lubricating eye drops (Zyaqua, Carboxymethyl cellulose sodium eye drops, 0.5% w/v) for one month for symptomatic relief. Along with this, she was advised to apply warm compresses to the eyelids for several minutes, two to four times daily. She was counselled to take complete rest, to avoid any cosmetics and not to treat any patients till the symptoms resolve. After 4 days of the treatment, the eyelid swelling got resolved and there was no pain and redness. After a week, she treated another patient for direct pulp capping procedure and encountered similar infection again, but the severity of the infection was in a milder form. The right eye showed nodular swelling at the medial margin of the upper lid with normal anterior segment (Fig. 2). There was no associated redness or pain present. The patient was advised to continue the lubricating eye drops. She was informed to report back to the ophthalmologist if the symptoms persist. After a month of treatment, she had no redness, no eyelid swelling or pain and had complete recovery.

## Discussion

Dental procedures often cause ocular injury due to combination of insults which can be microbial, physical, chemical, etc. Both the dentist and patient are at risk due to the use of power-driven handpieces which generate aerosols. Operative procedures like caries removal use dental handpieces on a frequent basis which release a lot of aerosol matter into the atmosphere. The most likely cause for the eyelid swelling was microbial infection present in the carious lesion which was transmitted via the aerosols generated by the airotor during the cavity cutting procedure [3, 6, 7].

Various surveys including those by Ramos MF [8], Stokes AN [9] have emphasized that the awareness and eye protection for the dental staff is not up to the standards. Most often, the particulate matter is lodged in the cornea or conjunctival sac which causes irritation and redness. In certain instances, serious eye injuries like perforation, irritation of lens might occur due to the particle getting penetrated into deeper tissues [10, 11]. Eyes are vital and delicate structures, and hence they are easily affected with the infectious matter like aerosols without any contact [3]. Once contacted, the infection can range from a mild to severe swelling to serious complications like retinal damage, or formation of scars and ulcers which can cause obstruction of vision [12]. In this case, though the clinician was wearing her normal custom spectacles, it was not enough to prevent the infection from happening. This suggests extra that precautionary measures while dealing with such cases should be recommended. A study by B.A.Aydil et al. [13] demonstrated that ocular injuries reported were significantly at a higher rate among the participants without any eye protection and also suggested that there were major inadequacies in the eye/face protection protocols.

With the present pandemic situation, eye protection is must and mandatory. It is interesting to note that on 15th March 2020, *The New York Times* in their paper published that dentists are the highly risked and exposed healthcare workers of being affected by COVID-19 [14]. In the course of dental procedures, aerosol inhalation generated by the instruments when working on COVID-19 patients is considered as high risk [15]. Despite the virus transmission routes, it is advised to adopt protective glasses and visors as safe and careful approach when performing the dental procedures [16].

**Fig. 3:**
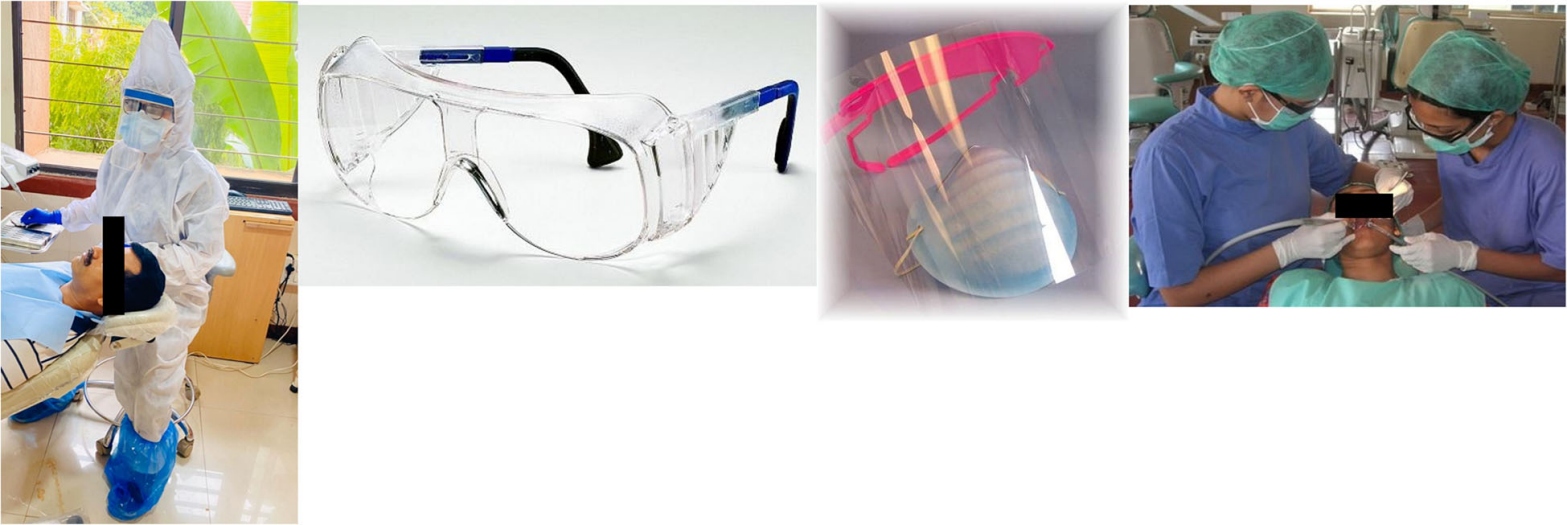
Protective eyewear with side shields and face shields can protect the eyes from spatter or debris generated during dental procedures

ADA and OSHA have demarcated the use of designated protective eyewear with side shield and the use of face shields while performing dental procedures (Fig. 3). OSHA recommended the use of glasses which meet OSHA Standard 1910.133(a) (1) and must meet ANSI Standard (Z87.1) to prevent the frontal entry route of the debris. They also highlighted the use of side shields which meet OSHA Standard 1910.133(a) for the prevention of debris travelling sideways (www.osha.gov/SLTC/etools/eyeandface/ppe/impact.html). OSHA also suggested the use of bottom gaps in the eyewear to prevent the travelling of debris vertical and tangential to the face [17]. They also outlined the implementation of eye wash station within 7.62 m in the vicinity [5]. The awareness and knowledge about ocular injuries and the need for proper precaution should be emphasized at the undergraduate level and should be highlighted in all the clinical work-stations.

## Conclusion

This case report describes acute infection of the eyelid secondary to allergic reaction due to the restorative dental procedures. Ocular injuries can be minimized by the application of standard guidelines. This further spotlights the importance of additional awareness and implementation of protective protection equipment such as a face shield along with protective eye wear.

## Data Availability

Data sharing is not applicable to this article as no datasets were generated or analysed during the current study.

## Abbreviations

OSHA: Occupational Safety and Health Administration
ANSI: American National Standard Institute
CDC: Centers for Disease Control and Prevention
ADA: American Dental Association
